# Combined effects of *IL-8 *and *CXCR2 *gene polymorphisms on breast cancer susceptibility and aggressiveness

**DOI:** 10.1186/1471-2407-10-283

**Published:** 2010-06-12

**Authors:** Kaouther Snoussi, Wijden Mahfoudh, Noureddine Bouaouina, Meriem Fekih, Hedi Khairi, Ahmed N Helal, Lotfi Chouchane

**Affiliations:** 1 Laboratoire d'Immuno-Oncologie Moléculaire, Faculté de Médecine de Monastir, Université de Monastir, Monastir, 5019, Tunisia; 2 Départment de Cancérologie Radiothérapie, CHU Farhat Hached, Sousse, 4000, Tunisia; 3 Service d'Obstétrique et des maladies féminines, Centre Hospitalo-Universitaire-Farhat-Hached de Sousse, Sousse, 4000, Tunisia; 4 Unité Génome, Diagnostic Immunitaire et Valorisation, Institut Supérieur de Biotechnologie de Monastir, Université de Monastir, Monastir, 5000, Tunisia; 5 Department of Genetic Medicine, Weill Cornell Medical College in Qatar, P.O.Box 24144, Doha, Qatar

## Abstract

**Background:**

Interleukin-8 (IL-8/CXCL-8) is a prototype of the ELR^+^CXC chemokines that play an important role in the promotion and progression of many human cancers including breast cancer. We have recently showed the implication of polymorphism (-251) T/A of *IL-8 *gene in the susceptibility and prognosis of breast carcinoma. IL-8 acts through its CXCR1 and CXCR2 receptors. CXCR2, expressed on the endothelial cells, is the receptor involved in mediating the angiogenic effects of ELR^+^CXC chemokines and in particular IL-8.

In the current study, we investigated the susceptibility and prognostic implications of the genetic variation in CXCR2 in breast carcinoma. We also confirmed the implication of *IL-8 (-251) T/A *polymorphism in a larger cohort. Finally, we combined the *IL-8 *and *CXCR2 *variant alleles and analyzed their effects in breast cancer risk and prognosis.

**Methods:**

We used the allele-specific polymerase chain reaction to characterize the variation of *IL-8 *and *CXCR2 *for 409 unrelated Tunisian patients with breast carcinoma and 301 healthy control subjects. To estimate the relative risks, Odds ratios and 95% confidence intervals were calculated using unconditional logistic regression after adjusting for the known risk factors for breast cancer. Associations of the genetic marker with the rates of breast carcinoma-specific overall survival and disease-free survival were assessed using univariate and multivariate analyses.

**Results:**

A highly significant association was found between the homozygous *CXCR2 *(+ 1208) TT genotype (adjusted OR = 2.89; *P *= 0.008) and breast carcinoma. A significantly increased risk of breast carcinoma was associated with *IL-8 *(-251) A allele (adjusted OR = 1.86; *P *= 0.001). The presence of two higher risk genotypes (the TA and TT in *IL-8*, and the TT in *CXCR2*) significantly increased the risk of developing breast carcinoma (adjusted OR = 4.15; *P *= 0.0004).

The *CXCR2 *(+ 1208) T allele manifested a significant association with an aggressive phenotype of breast carcinoma as defined by a large tumor size, a high histological grade, and auxiliary's lymph node metastasis. A significant association between the *IL-8 *(-251) A allele and the aggressive form of breast carcinoma was also found.

Moreover, the presence of the *IL-8 *(-251) A and/or the *CXCR2 *(+ 1208) T allele showed a significant association with a decreased overall survival and disease-free survival in breast carcinoma patients.

**Conclusion:**

Our results indicated that the polymorphisms in *IL-8 *and *CXCR2 *genes are associated with increased breast cancer risk, as well as disease progress, supporting our hypothesis for IL-8 and ELR^+^CXC chemokine receptor (CXCR2) involvement in breast cancer pathogenesis.

## Background

Breast cancer is one of the most prevalent cancers in the world. Despite progress made in the last 30 years in breast cancer screening and treatment, this disease is still responsible for almost half a million deaths per year worldwide. Approximately half of diagnosed patients will eventually develop metastatic disease. Treatment for metastatic breast cancer is palliative, and median life expectancy after recurrence is between 24 and 30 months or less [1,2].

The etiology of breast cancer is extremely complex and, while not yet elucidated, appears to involve numerous genetic, endocrine and external environmental factors. The role of genetic factors in epidemiology and pathogenesis of both sporadic and familial breast cancer is now well established. Only a small minority (~5%) of patients with breast cancer develop the disease as a result of inheritance of germline mutations in dominant, highly penetrant susceptibility genes such as *BRCA1 *and *BRCA2*. However, polymorphisms in the genes involved in the complex mechanisms of carcinogenesis may confer low penetrant susceptibility to breast cancer in a significant proportion of the remaining patients [3].

The neoplastic transformation, growth, survival, invasion, and metastases are dependent on the establishment of a pro-angiogenic environment. Local angiogenesis is determined by an imbalance in the over-expression of pro-angiogenic factors, as compared to inhibitors of angiogenesis. The CXC chemokine family is the unique group of cytokines known for their ability to behave in a disparate manner in angiogenesis regulation. Several members of the CXC chemokine are potent promoters of angiogenesis, whereas others inhibit the angiogenic process. The disparity in angiogenic activity among CXC chemokine family members is attributed to three amino acid structural domains at the N terminus, Glu-Leu-Arg (ELR), which is present in angiogenic (i.e., CXCL1, CXCL2, CXCL3, CXCL5, CXCL6, CXCL7, and CXCL8) [4-6], but not angiostatic (i.e., CXCL4, CXCL9, CXCL10, and CXCL11) CXC chemokines [7].

ELR^+ ^CXC chemokines play an important role in tumor growth and progression in a number of tumor model systems [8]. In particular, interleukin-8 (IL-8/CXCL8), which was originally described as a leukocyte chemoattractant [9], was subsequently found to possess mitogenic and angiogenic properties [10,11]. Several studies suggested a tendency toward the involvement of IL-8 in cancer development [12]. Elevated IL-8 levels were associated with disease progression and recurrence in human prostate, lung, gastric, and breast cancers [13-16].

All angiogenic ELR^+ ^CXC chemokines mediate their angiogenic activity through CXCR2 [17]. Subsequent studies have confirmed the expression of CXCR2, not CXCR1, to be the primary functional chemokine receptor in mediating endothelial cell chemotaxis [18,19]. By considering the expression of IL-8 by breast cancer cells and CXCR2 by large vessel and microvascular endothelial cells [19,20], an autocrine effect for IL-8 and the chemokine receptor CXCR2 has been suggested.

Promoter regions of a number of cytokine genes contain polymorphisms that directly influence cytokine production [21]. The *IL-8 *gene is located on chromosome 4q13-21 and consists of four exons, three introns, and a proximal promoter region [22]. Several polymorphisms have been reported in the *IL-8 *gene. Interestingly, IL-8 production can be controlled by the -251 A/T in the promoter region of this chemokine [23]. Recent data revealed that the *IL-8 *(-251) A allele is associated with a high expression level of IL-8 protein and a severe neutrophil infiltration [23].Other studies also reported that the *IL-8 *(-251) T/A polymorphism is associated with a higher risk of developing malignant diseases [24-27].

Three single polymorphisms at positions +785 C/T, +1208 T/C and +1440 G/A were reported in the *CXCR2 *gene [28]. Several reports indicated that the polymorphism +1208 C/T which is located in the non-coding region of *CXCR2 *gene might provide valuable information for the pathogenesis and the susceptibility to chronic inflammatory disease [28,29].

Previously we have reported an elevated risk for breast cancer among the Tunisian population (*n *= 308 cases and *n *= 236 healthy controls) associated with the *IL-8 *(-251) T/A polymorphism. The implication of this polymorphism in the prognosis of breast carcinoma has also been demonstrated [30].

Based on the abundant evidence for the role of IL-8 and CXCR2 in carcinogenesis, we evaluated in this study the association of *CXCR2 *(+ 1208) C/T gene polymorphism and breast cancer susceptibility and prognosis in Tunisia. Then we used existing and additional genotype data on 409 breast cancer cases and 301 healthy controls to examine the potential contribution of the combined genotypes of *IL-8 *and *CXCR2 *in breast carcinoma occurrence, clinico-pathological characteristics and prognosis.

## Methods

### Patients and controls

*IL-8 *and *CXCR2 *genotype and allele frequencies were determined in a group of 301 control subjects and 409 patients with breast carcinoma. Controls and patients were selected from the same population living in the middle coast of Tunisia and including unrelated subjects.

Data on patient, tumour and treatment characteristics at the study entry for each subject were collected from the department of Radiation Oncology and Medical Oncology of Sousse Hospital (Sousse, Tunisia) between 1996 and 2004. They were selected consecutively whenever practically feasible.

All patients included in this study had primary breast carcinoma, with unilateral breast tumours and with no family history for the disease. The diagnosis of cancer was confirmed by histopathological analyses. The patients (*n = *409) had a mean age of 48.0 ± 24.3 (SD) years. At time of analysis, 88 patients experienced recurrence (local or distant). Among them, 56 died from breast carcinoma (63.64%). Table 1 shows the treatment description of all patients. A total of 301 healthy women having a mean age of 48.0 ± 14.9 years, were blood donors with no evidence of any personal or family history of cancer (or other serious illness). Samples from healthy controls were collected consecutively between 1996 and 2004. Control individuals were frequency matched to the expected age distribution of the cases and were from the same geographical area. General characteristics of breast cancer patients and healthy controls women are summarized in Table 1.

**Table 1:** The general characteristics of breast cancer patients and healthy controls from Tunisian women blood donors

**Characteristics, n (%)**^**a**^	Cases	Controls	***P*-value**^**b**^
**Age^b^, yr**			
Mean ± SD	48.0 ± 24.	48.0 ± 14.9	0.97
**Body mass index (BMI, kg/m^2^)**	24.1 ± 5.4	20.6 ± 2.5	0.0004
**History of benign breast disease**			
No	241 (58.92)	154 (45.18)	
Yes	168 (41.08)	147 (54.82)	0.039
**Use of oral contraceptive**			
No	143 (34.96)	141 (46.84)	
Yes	266 (65.04)	160 (53.16)	0.001

^a ^Cases: n = 409; Controls: n = 301. The percentages were shown in the parentheses

^b ^Age and BMI were determined by two-sample t tests; history of benign breast disease and oral contraceptive drugs usage were determined by chi-square tests.

A detailed description of the clinico-pathological characteristics of breast cancer cases has been reported elsewhere [31] and data on tumor size at diagnosis, nodal status and histological grade are briefly included in Table 2.

**Table 2:** Clinicopathologic Characteristics of the 409 Breast Carcinoma and the Corresponding Univariate Analysis of death (OVS) and Reccurence^a ^(DFS)

		Breast carcinoma specific OVS		DFS	
**Characteristic**	**%**	**6-year rate**	***P*-value**	**6-year rate**	***P*-value**

**Clinical tumor size**					
T1-T2	67.08	82.85		70	
T3-T4	32.92	62.86	< 0.001	32.86	< 0.000001
**Lymph node status**^**b**^					
N (+)	46.72	84.23		71.43	
N (-)	53.28	68.57	< 0.01	44.28	< 0.00001
**SBR grading**					
1-2	60.87	79.28		69.10	
3	40.13	57.28	< 0.02	47.80	< 0.01
**Age (yrs)**					
< 50	57.32	78.1		70.0	
≥50	42.68	75.71	NS	67.2	NS

OVS: overall survival; DFS: disease free survival; SBR: Scarff, Bloom and Richardson; NS: not significant.

^a^Six-year survival rates were estimated according to Kaplan and Meier. The log rank test was used to determine whether significant differences (*P *value) were observed between subgroups of patients.

^b^Determined based on the pathologic examination.

Both patients and controls gave their written consent to participate in the study and to allow their biological samples to be genetically analyzed. Approval for the study was given by the National Ethical Committee.

### Genomic DNA Extraction

Genomic DNA was extracted from peripheral blood leukocytes by a salting procedure [32]. Briefly, 10 ml of blood was mixed with triton lysis buffer (0.32 M sucrose, 1% Triton X-100, 5 mM MgCl_2_, H_2_O, 10 mM Tris-HCl, pH 7.5). Leukocytes were spun down and washed with H_2_O. The pellet was incubated with proteinase K at 56°C and subsequently salted out at 4°C using a substrate NaCl solution. Precipated proteins were removed by centrifugation. The DNA in supernatant fluid was precipated with ethanol. The DNA pellet was dissolved in 400 μl H_2_O.

### IL-8 (-251) T/A and CXCR2 (+1208) C/T genotyping

An allele-specific polymerase chain reaction (AS-PCR) was used to detect the polymorphisms at positions -251 of *IL-8 *gene and +1208 of *CXCR2 *gene. For *CXCR2 *genotyping, 10 μl of PCR reaction mixture consisting of 50 ng of genomic DNA, 0.01 mM dNTPs, 2 mM MgCl_2_, 1× Taq polymerase buffer, 0.75 μM of each specific/common primer (Amersham, Paris, France), 0.2 μM of each internal control primer (see Table 3 for primer sequences) and 0.5 U of Taq DNA polymerase (Amersham, Paris, France) were employed. As an internal control, the Beta-globin specific primers were included in the AS-PCR (Table 2). First, a touch-down procedure 25 s at 95°C was carried out. This was followed by annealing for 45 s at temperatures decreasing from 66°C (four cycles) to 60°C (25 cycles) and an extension step at 72°C for 40 s. The genotypes of IL-8 (-251) T/A marker were determined as previously reported [30].

**Table 3:** Primer sequences used for genotyping of *IL-8 *(-251) T/A and *CXCR2 *(+1208) C/T gene polymorphisms (rs 4073 and rs 1801032)

Primer name	Primer sequence
*IL-8 *(-251) Common	5'-AAT ACG GAG TAT GAC GAA A-3'
*IL-8 *(-251) T (low expresser)	5'-CTA GAA ATA AAA AAG CAT ACA T-3'
*IL-8 *(-251) A (high expresser)	5'-CTA GAA ATA AAA AAG CAT ACA A-3'
HGH I CONTROL	5'-GCC TTC CCA ACC ATT CCC TTA-3'
HGH II CONTROL	5'-TCA CGG ATT TCT GTT GTG TTT-3'
*CXCR2 *(+1208) Common	5'-GTC TTG TGA ATA AGC TGC TAT GA-3'
*CXCR2 *(+1208) C Allele	5'-CCA TTG TGG TCA CAG GAA GC-3'
*CXCR2 *(+1208) T Allele	5'-CCA TTG TGG TCA CAG GAA GT-3'
Beta Globin I	5'-ACA CAA CTG TGT TCA CTA-3'
Beta Globin II	5'-CAA CTT CAT CCA CGT TCA CC-3'

HGH I and II primers amplify HGH (human growth hormone) sequence.

The reaction products of *IL-8 *and *CXCR2 *gene amplification were loaded onto 2% agarose gel containing ethidium bromide, electrophoresed and visualized under UV transillumination. To ensure quality control of all genotyping results, 25% of randomly selected samples of both cases and controls were analysed a second time, without finding any discrepancies.

### Statistical analyses

The genotype and allele frequencies of *IL-8 *and *CXCR2 *were tested for the Hardy-Weinberg equilibrium for both patient and control groups using the chi-square test. Two-sample t tests and Chi-square tests were used to explore the bivariate association between the status of breast cancer and other covariates for continuous and categorical variables, respectively. Risk association between the genotypes and breast cancer susceptibility and tumours characteristics was estimated by odds ratio (OR) and 95% confidence intervals (95% CI) using multivariate logistic regression analysis. The model for adjusted OR included age at diagnosis, history of benign breast disease, use of oral contraceptive, and body mass index (BMI).

Disease-free survival (DFS) was defined as the time from the date of diagnosis to the first local or distant recurrence or to last contact. Breast carcinoma-specific overall survival (OVS) was defined as the time from the date of diagnosis to death if the patient died from breast carcinoma or to last contact. Six-year survival rates were estimated, and survival curves were plotted according to Kaplan and Meier [33]. The differences between groups were calculated by the log-rank test [34].

In multivariate analysis, relative risk of recurrence or death from breast carcinoma, 95% confidence intervals, and *P *values for censored survival data were calculated by use of Cox proportional hazards regression Model [35]. All *P *value calculations were two-sided, and *P *value was considered significant at less than 0.05. Only clinicopathologic parameters bearing prognostic significance were included in the Cox model. Clinicopathological parameters were dichotomised as follows: nodal status (≥1 *versus *no positive lymph node), SBR (Scarff, Bloom and Richardson) tumour grade (1-2 *versus *3), clinical tumour size (T_1_-T_2 _*versus *T_3_-T_4_).

The statistical analysis was performed using SEM-STATISTIQUES software (centre Jean Perrin, Clermont-Ferrand, France).

## Results

### Polymorphisms in the IL-8 and CXCR2 genes as risk factors for breast carcinoma

The breast cancer patients (n = 409) and healthy controls (n = 301) were all native Tunisian women. The characteristics of the healthy controls and breast cancer patients, including overage ages, body mass index (BMI), history of benign breast disease, and oral contraceptives usage were summarized in Table 1. There were no significant differences between cases and controls concerning age. However, BMI, history of benign breast disease, and oral contraceptives usage were significantly different between cases and controls after the statistical testing (see materials and methods). These confounding factors were adjusted in multivariate logistic regression analysis.

The genotype distribution and allele frequencies for the *IL-8 *(-251) T/A and *CXCR2 *(+1208) C/T polymorphisms in all breast carcinoma patients and controls are presented in Table 4. The allele frequencies of *IL-8 *and *CXCR2 *genes were in Hardy-Weinberg equilibrium in both patients and controls (*P *= , *P *= , *P *= , *P *= respectively).

**Table 4:** The *IL-8 *(-251) T/A and *CXCR2 *(+1208) C/T genotype distributions in Control Subjects and in Patients with Breast Carcinoma

Genotypes	Patients (n = 409)	Controls (n = 301)	Crude OR (95% CI)	***P*-value**^**a**^	**Adjusted OR (95% CI)**^**b**^	*P*-value
	*n *(%)	*n *(%)				
***IL-8 *(-251)T/A**						
TT	84 (20.5)	92 (30.6)	1		1	
TA	201 (49.2)	138 (45.8)	1.60 [1.09-2.34]	0.01	1.71 [1.13-2.55]	0.009
AA	124 (30.3)	71 (23.6)	1.91 [1.24-2.96]	0.002	2.03 [1.56-3.67]	0.001
**Alleles**						
T-allele	369 (45.1)	322 (53.5)				
A-allele	449 (54.9)	280 (46.5)	1.40 [1.13-1.74]	0.001	1.86 [1.79-2.45]	0.001

***CXCR2 *(+1208)C/T**						
CC	195 (46.7)	155 (51.5)	1		1	
CT	167 (40.8)	128 (42.5)	1.04 [0.75-1.43]	0.81	1.31 [0.90-1.69]	0.63
TT	47 (11.5)	18 (6.0)	2.08 [1.12-3.88]	0.01	2.89 [1.48-4.55]	0.008
**Alleles**						
C-allele	557 (6.81)	438 (7.28)				
T-allele	261 (31.9)	164 (27.2)	1.25 [0.99-1.59]	0.05	1.37 [1.09-1.88]	0.03

^a ^*P*-value determined by χ^2 ^test.

^b ^ORs were adjusted for age, BMI, history of benign disease, and oral contraceptive drugs usage.

A significantly higher risk for breast cancer was observed for carriers of *IL-8 *(-251) AA genotype (adjusted Odds Ratio (OR) = 2.03; *P *= 0.001) and carriers of *IL-8 *(-251) TA genotype (OR = 1.71; *P *= 0.009). The *IL-8 *(-251) A allele was significantly higher in patients compared to controls (OR = 1.86; *P *= 0.001).

The genotype frequency of the *CXCR2 *(+1208) TT was 0.115 in patients with breast carcinoma and 0.06 in control subjects (OR = 2.89; *P *= 0.008). The *CXCR2 *(+1208) T allele was significantly higher in patients compared to controls (OR = 1.37; *P *= 0.03) (Table 4).

Because IL-8 functionally interacts with CXCR2 we assessed the effect of multiple genotypes on breast carcinoma risk. Through individual investigations of genotypes, we defined *IL-8 *(-251) TA, *IL-8 *(-251) AA, and *CXCR2 *(+1208) TT as high-risk genotypes.

Table 5 shows the relation between ORs and the number of high-risk genotypes. In controls, there were no high-risk genotypes in 26.91%, 1 in 68.44% and 2 in 4.65%. However, in breast cancer patients, there were no high-risk genotypes in 18.3%, 1 in 71.7% and 2 in 10.02%. The presence of 1 or 2 high-risk genotypes significantly increased the risk of developing breast carcinoma, with ORs of 1.63 (95% CI, 1.13 - 2.59; *P *= 0.01) and 4.15 (95% CI, 1.92 - 7.32; *P *= 0.0004), respectively, compared with the absence of the high-risk genotype.

**Table 5:** Interaction and addictive effects of *IL-8 *(-251) T/A and *CXCR2 *(+1208) C/T polymorphisms on breast cancer risk

Genotypes	Patients (n = 409)	Controls (n = 301)	Crude OR (95% CI)	***P*-value**^**a**^	**Adjusted OR (95%)**^**b**^	*P*-value
***IL-8 *(-251)*/CXCR2 *(+1208)**	*n *%	*n *%				
**0**	75 (18.34)	81 (26.91)	1		1	
**1**	293 (71.64)	206 (68.44)	1.54 [1.05-2.24]	0.01	1.63 [1.13-2.59]	0.01
**2**	41 (10.02)	14 (4.65)	3.16 [1.52-6.64]	0.0006	4.15 [1.92-7.32]	0.0004

^a ^*P*-value determined by χ^2 ^test.

^b ^ORs were adjusted for age, BMI, history of benign disease, and oral contraceptive drugs usage.

The high risk genotypes: *IL-8 *(-251) T/A = TA/AA; *CXCR2 *(+1208) C/T = TT

### Prognostic significance of polymorphism in IL-8 and CXCR2 genes

Table 2 shows the clinicopathological characterization. The distribution of the clinicopathological markers was in accordance with previously reported data, indicating that our cohort was representative of breast carcinoma patients. Disease-free survival and breast carcinoma-specific OVS rates were estimated and compared by univariate analysis on these clinicopathological parameters. Significant associations were found for clinical tumor size, lymph node status, and tumor grading with DFS and OVS. No significant differences were observed for age.

The distributions of *IL-8 *and *CXCR2 *polymorphisms according to the clinico- pathological indices of breast carcinoma severity are presented in Tables 6 and 7. A significant association between *IL-8 *(-251) A allele and large tumor size (T3-T4), high SBR tumor grade (grade 3), and lymph node metastases was observed (Table 6). In this analysis, we also observed the association of this polymorphism with the hormone status of breast carcinoma. Interestingly, a pronounced association was found between the *IL-8 *(-251) A allele and a negative hormone status (OR = 1.82; *P *= 0.0008).

**Table 6:** Genotype frequencies of *IL-8 *(-251) T/A polymorphism in relation to pathological indices of Breast Cancer severity

Genotypes	Number of patients (%)		Crude OR (95% CI)	***P-*value**^***a***^	**Adjusted OR (95% CI)**^**b**^	*P*-value
**Clinical tumor size**	**T1-T2**	**T3-T4**				
TT	65 (27.08)	37 (24.51)	1		1	
TA	122 (50.84)	58 (38.41)	0.84 [0.49-1.44]	0.48	0.74 [0.67-1.32]	0.87
AA	53 (22.08)	56 (37.08)	1.86 [1.03-3.35]	0.02	1.79 [1.11-3.76]	0.01
**Alleles**						
T-allele	252 (52.50)	132 (43.71)				
A-allele	228 (47.50)	170 (56.29)	1.42 [1.05-1.92]	0.01	1.57 [1.32-2.05]	0.01
**Lymph nodes status**	**Negative**	**Positive**				
TT	50 (27.62)	42 (19.91)	1		1	
TA	92 (50.83)	95 (45.02)	1.46 [0.85-2.52]	0.14	1.32 [0.78-2.01]	0.21
AA	39 (21.55)	74 (35.07)	2.26 [1.24-4.14]	0.004	2.59 [1.47-5.32]	0.002
**Alleles**						
T-allele	192 (53.04)	179 (42.42)				
A-allele	170 (46.96)	243 (57.58)	1.53 [1.14-2.05]	0.002	1.58 [1.28-2.24]	0.001
**SBR grading**	**1-2**	**3**				
TT	66 (32.19)	46 (28.22)	1		1	
TA	111 (54.15)	67 (41.11)	0.87 [0.52-1.45]	0.55	0.91 [0.58-1.49]	0.34
AA	28 (13.66)	50 (30.67)	2.56 [1.35-4.87]	0.001	2.69 [1.52-5.24]	0.0009
**Alleles**						
T-allele	243 (59.27)	159 (48.77)				
A-allele	167 (40.73)	167 (51.23)	1.53 [1.13-2.07]	0.004	1.66 [1.38-2.79]	0.002
**Estrogen receptor status**	**Negative**	**Positive**				
TT	41 (23.7)	51 (41.13)	1		1	
TA	75 (43.35)	44 (35.48)	2.12 [1.17-3.84]	0.007	2.01 [1.32-3.76]	0.006
AA	57 (32.95)	29 (23.39)	2.44 [1.28-4.70]	0.003	2.56 [1.68-4.96]	0.001
**Alleles**						
T-allele	157 (45.38)	146 (58.87)				
A-allele	189 (54.62)	102 (41.13)	1.72 [1.22-2.48]	0.001	1.82 [1.96-2.78]	0.0008

^a ^*P*-value determined by χ^2 ^test.

^b ^ORs were adjusted for age, BMI, history of benign disease, and oral contraceptive drugs usage.

Furthermore, the frequency of the *CXCR2 *(+1208) T allele was significantly higher in patients with a large tumor size (OR = 1.98; *P *= 0.0001), with high SBR tumor grade (grade 3) (OR = 1.67; *P *= 0.01), and with lymph node metastases (OR = 1.83; *P *= 0.0008). Taken together, these results suggest that the (-251) A allele of the *IL-8 *gene and the *(+1208) T *allele of the *CXCR2 *gene are associated with the aggressive forms of breast carcinoma. No association was found between *CXCR2 *gene polymorphism and the hormone status of breast cancer patients (Table 7).

**Table 7:** Genotype frequencies of *CXCR2 *(+1208) C/T polymorphism in relation to pathological indices of Breast Cancer severity

Genotypes	Number of patients (%)		Crude OR (95% CI)	***P-*value**^***a***^	**Adjusted OR (95% CI)**^**b**^	*P*-value
**Clinical tumor size**	**T1-T2**	**T3-T4**				
CC	117 (48.75)	51 (33.55)	1		1	
CT	89 (37.08)	62 (40.79)	1.60 [0.98-2.61]	0.04	1.73 [1.01-2.76]	0.03
TT	34 (14.17)	39 (25.66)	2.63 [1.44-4.82]	0.0006	2.89 [1.64-5.06]	0.0004
**Alleles**						
C-allele	323 (67.29)	164 (53.95)				
T-allele	157 (32.71)	140 (46.05)	1.76 [1.29-2.39]	0.0001	1.98 [1.47-2.56]	0.0001

**Lymph nodes status**	**Negative**	**Positive**				
CC	94 (51.93)	81 (38.39)	1		1	
CT	69 (38.12)	89 (42.18)	1.50 [0.95-2.36]	0.06	1.45 [0.98-2.27]	0.07
TT	18 (9.95)	41 (19.43)	2.64 [1.35-5.21]	0.002	2.72 [1.44-5.43]	0.001
**Alleles**						
C-allele	257 (70.99)	251 (59.48)				
T-allele	105 (29.01)	171 (40.52)	1.67 [1.22-2.27]	0.0007	1.83 [1.32-2.46]	0.0008

**SBR grading**	**1-2**	**3**				
CC	91 (44.39)	63 (38.18)	1		1	
CT	85 (41.46)	64 (38.78)	1.09 [0.67-1.76]	0.71	1.12 [0.78-1.89]	0.65
TT	29 (14.15)	38 (23.03)	1.89 [1.02-3.53]	0.03	1.98 [1.15-3.79]	0.02
**Alleles**						
C-allele	267 (65.12)	190 (57.58)				
T-allele	143 (34.88)	140 (42.42)	1.38 [1.01-1.87]	0.03	1.67 [1.08-2.03]	0.01

**Estrogen receptor status**	**Positive**	**Negative**				
CC	50 (30.87)	48 (33.33)	1		1	
CT	76 (46.91)	60 (41.67)	1.22 [0.70-2.12]	0.46	1.34 [0.78-2.34]	0.35
TT	36 (22.22)	36 (25.00)	0.96 [0.50-1.85]	0.89	0.87 [0.54-1.89]	0.85
**Alleles**						
C-allele	176 (54.32)	156 (54.17)				
T-allele	148 (45.68)	132 (45.83)	0.99 [0.71-1.38]	0.96	0.95 [0.66-1.27]	0.87

^a ^*P*-value determined by χ^2 ^test.

^b ^ORs were adjusted for age, BMI, history of benign disease, and oral contraceptive drugs usage.

When the relationship between the distribution of *IL-8 *and *CXCR2 *genotypes in all patients and the survival (OVS and DFS) was tested, significant differences were observed between the OVS and the DFS Kaplan-Meier survival curves for the different polymorphisms.

**Figure 1:**
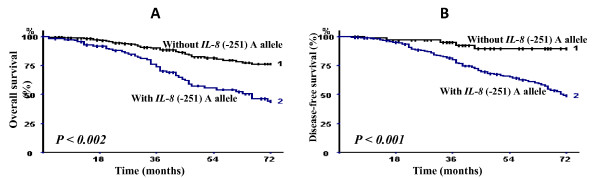
**Breast carcinoma-specific overall survival (A) and disease-free survival (B) of 409 breast carcinoma patients according to the presence or absence of *IL-8 *(-251) A allele (*P *denotes the log-rank test value)**.

The breast carcinoma-specific OVS was significantly shorter among patients carrying the *IL-8 (-251) A *allele (Figure 1A). The estimated 3- and 6-year breast carcinoma-specific OVS rates for the group of patients carrying or not carrying the *IL-8 *(-251) A allele were respectively 91.4 and 77.1% *versus *75.7 and 45.7% (log rank test, *P *< 0.002). The estimated 3- and 6-year DFS rates in the group of patients with *IL-8 *(-251) A allele were 81.4% and 50% *versus *95.7% and 88.5% in the group of patients without the *IL-8 *(-251) A allele (log rank test, *P *< 0.001).

**Figure 2:**
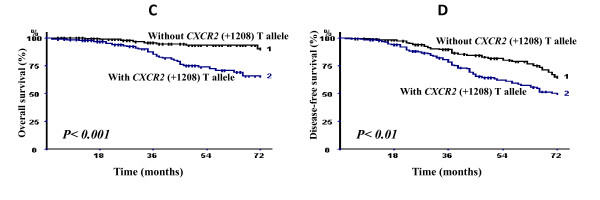
**Breast carcinoma-specific overall survival (C) and disease-free survival (D) of 409 breast carcinoma patients according to the presence or absence of *CXCR2 *(+1208) T allele (*P *denotes the log-rank test value)**.

As shown in Figure 2, the breast carcinoma specific OVS and DFS were significantly shorter in the group of patients carrying *CXCR2 *(+1208) T allele. The estimated 3- and 6-year breast carcinoma OVS rate in the group of patients carrying *CXCR2 *(+1208) T allele were, respectively, 87.1 and 65.7% *versus *95.7% and 90% for those not carrying the *(+1208) T *allele (log rank test, *P *< 0.001). The estimated 3- and 6-year DFS rates in the group of patients with *CXCR2 *(+1208) T allele were 74.3% and 50% *versus *90% and 61.4% in the group of patients without the *CXCR2 *(+1208) T allele (log rank test, *P *< 0.01).

Multivariate analyses were undertaken to evaluate the importance of *IL-8 *and *CXCR2 *markers in the recurrence risk and death compared with the clinicopathological parameters. Introducing the genetic and the clinicopathological parameters bearing prognostic significance we tested the Cox model. No genetic and clinicopathological parameters were selected for OVS and DFS.

Further analyses were conducted to explore whether combined high-risk genotypes of the *IL-8 *and *CXCR2 *genes were associated with clinicopathological indices and survival among patients. No additional effect was found between the presence of more than one high-risk genotype and indices of an aggressive form of breast carcinoma (nodal status, SBR, tumor grade) or OVS and DFS (data not shown).

## Discussion

The ELR^+ ^CXC chemokines play an important role in tumor growth and progression in a number of tumor model systems. IL-8/CXCL8 was the first described angiogenic, mitogenic, and motogenic chemokine in various cancer models and is the prototype of ELR^+ ^CXC chemokines [8,10-13]. This chemokine was initially discovered on the basis of its ability to induce mobilization of neutrophils and lymphocytes *in vivo *[9]. Like the basic fibroblast growth factor (bFGF) and the vascular endothelial growth factor (VEGF), it is a strong angiogenesis inducer. IL-8 mediates endothelial cell chemotaxis and proliferation *in vitro *and *in vivo *[36].

The fact that all ELR^+^CXC chemokines mediate angiogenesis highlights the importance of identifying a common receptor that mediates their biological functions in promoting angiogenesis. The candidate CXC chemokine receptors are CXCR1 and CXCR2. Only CXCL-8/IL-8 and CXCL-6 specifically bind to CXCR1 whereas all ELR^+^CXC chemokines bind to CXCR2. There is evidence that CXCR2 is implicated in the angiogenic activity of ELR^+ ^CXC chemokines [18,36].

In recent years, several studies have shown that IL-8 and CXCR2 are overexpressed in a range of human cancers including renal, prostate, pancreatic, colon, nasopharyngeal, and gastric cancers [37-41]. IL-8 and its receptors were detected on breast tumor cells and endothelial cells of tumor vessels [19-21]. In addition, IL-8 levels are significantly higher in breast cancer patients compared with healthy controls [42]. Ben-Baruch et al have demonstrated that, aside the role of IL-8 and CXCR2 in mediating the recruitment of the tumor-infiltrating leucocytes to tumor site, their expression may also affect neoplastic proliferation and metastasis [43].

IL-8 and CXCR2 overexpression may present a risk factor in the development and progression of solid tumors. Several polymorphisms have been identified in *IL-8 *and *CXCR2 *genes. Among these, *IL-8 *(-251) T/A polymorphism exerts one of the greatest influences on IL-8 production. Polymorphism +1208 C/T of the *CXCR2 *gene is implicated in the susceptibility to and the pathogenesis of chronic inflammatory diseases [28,29].

Recently, we have showed that *IL-8 *(-251) T/A polymorphism may be a genetic risk factor for breast cancer onset and severity in the Tunisian population [30]. Our current study aims to confirm these results in a larger cohort and to determine whether there is any association between the genetic polymorphism of the *CXCR2 *and both individual susceptibility to and prognosis of breast carcinoma. Based on the intertwined and interactive roles that IL-8 and CXCR2 play at the molecular level in the angiogenic pathway, we further hypothesized a priori that the joint effect of genetic variants in these angiogenesis regulators may increase breast cancer risk.

In the present study, the comparison of genotype frequencies of *IL-8 *for breast carcinoma patients and control subjects indicates an increase of *IL-8 *(-251) TA and AA genotypes. Consequently, the *IL-8 *(-251) A allele frequency was found to be significantly higher in patients compared with controls. These results confirmed our previous findings in a smaller subset of cases and controls [31].

*CXCR2 *genotype analysis revealed that carriers of *CXCR2 *(+1208) TT homozygous genotype are significantly over-represented among breast cancer cases (OR = 2.08; *P *= 0.01). Despite the great interest in CXCR2 biological properties, little is known about the functional importance of single nucleotide polymorphisms in its gene. This result indicates that *CXCR2 *gene polymorphism could be considered as a susceptibility gene in breast cancer development.

These findings suggested that individual genetic polymorphisms of *IL-8 *and *CXCR2 *were associated with breast carcinoma risk. However, the combination of these genotypes showed a marked association with breast carcinoma risk. We defined the TA and AA genotypes of the *IL-8 *gene and the TT genotype of the *CXCR2 *gene as high-risk genotypes according to the individual genotype analysis. Breast carcinoma risk significantly increased according to the number of high-risk genotypes. There was a 63% increase in breast cancer (OR = 1.63; *P *= 0.01) for the presence of one high-risk genotype. The risk was 4.15 (*P *= 0.0004) for individuals with two high-risk genotypes. These data, taken together, suggest that there is evidence of a gene-dosage effect.

The assessment of the prognostic value of *IL-8 *genetic marker in breast carcinoma confirmed the results of our previous study and indicated that *IL-8 *(-251) A allele is highly associated with aggressive forms of breast carcinoma as defined by large tumor size, high grade and lymph node metastases. In the current study, we also examined for the first time the relationship between *IL-8 *(-251) T/A polymorphism and the hormonal status. Interestingly, we observed a significant association between *IL-8 *(-251) A allele and a negative hormonal status (*P *= 0.0008).

Furthermore, we investigated the association of the *CXCR2 *(+1208) C/T polymorphism with markers of tumor progression. Our results showed a significant association between *CXCR2 *(+1208) T allele and a large tumor size (*P *= 0.0001), high SBR tumor grade (*P *= 0.01), and lymph node metastases (*P *= 0.0008). More interestingly, we showed that *IL-8 *(-251) A and *CXCR2 *(+1208) T alleles were associated with a shorter overall survival and disease-free survival and, therefore, with a poor prognosis in breast carcinoma.

Given the importance of IL-8 and CXCR2 in angiogenesis, we investigated the relationship between polymorphisms -251 T/A in *IL-8 *gene and (+1208) C/T in *CXCR2 *gene and breast carcinoma. The results show that these polymorphisms may be related to breast carcinoma development and progression.

In agreement with our findings, several studies reported a relationship between *IL-8 *(-251) T/A and *CXCR2 *(+1208) C/T genes polymorphisms and human cancer. *IL-8 *(-251) A allele, which resulted in higher IL-8 secretion was associated with an increased risk and poor prognosis of colorectal cancer, prostate cancer, and gastric cancer [24-26,44]. Similarly, we reported in a previous study that *IL-8 *(-251) A allele held a higher risk of nasopharyngeal carcinoma and was highly associated with aggressive forms and poor prognosis [27]. Regarding breast carcinoma, the current study confirmed our previous results obtained in a smaller cohort and is also in agreement with Kamali-Sarvestani et al. who reported that *IL-8 (-251) A *allele carriers had a significantly higher risk of breast carcinoma than non carriers in the Iranian population [45].

Recently, few studies have shown the relationship between *CXCR2 *gene polymorphisms and human cancer [46,47]. The *CXCR2 *gene polymorphisms were associated with pancreatic cancer but not with prostate and breast cancer [46,47]. Kamali-Sarvestini et al. have demonstrated that *CXCR2 *(+1208) C/T polymorphism is not associated with breast cancer [45]. This result is in contrast with our findings.

There is now convincing evidence that these correlations between *IL-8 *A allele and cancer risk result from an increased level of IL-8 protein, which may have an impact on cancer development and progression via the regulation of immune response and pathways of tumor angiogenesis.

IL-8 is an important chemoattractant that promotes inflammatory processes [9]. The IL-8 receptors CXCR1 and CXCR2 have been reported to be present in a variety of cell types including inflammatory cells, endothelial cells, and fibroblasts [46]. Consequently, IL-8 is a major contributing factor involved in the initiation and amplification of the inflammatory response via its receptors [48]. However, it is known that inflammation profoundly affects the development and progression of tumor and therefore, IL-8 might promote tumor cell proliferation by amplification of inflammation in the tumor microenvironnement via its receptors.

IL-8 proangiogenic effects additionally stem from its ability to inhibit the apoptosis of endothelial cells [49]. This inhibition is associated with increased levels of the anti-apoptotic factors Bcl-xl and Bcl-2 as well as with decreased levels of Bax. It was further shown that IL-8 stimulates increased endothelial cell mRNA expression of matrix metalloproteinases (MMPs) MMP-2 and -9 as well as increases gelatine activity [49]. These MMP activities are required for the proteolytic modifications of basements membranes and extracellular matrices during angiogenesis.

IL-8 was shown to act as an autocrine growth factor and to stimulate invasion and chemotaxis of many tumor cell types. The expression of IL-8 and the receptor of ELR^+^CXC chemokine CXCR2 in cancer have been evaluated in numerous studies. Overexpression of IL-8 is associated with increasing tumor stage, disease progression and recurrence in human bladder, prostate, breast, lung, gastric, hepatic cancers, and melanoma [11,14-16,43,50,51]. Recently, it has been shown that the ER-negative breast cancer cells overexpressed IL-8. Concerning IL-8 receptors, it has been observed that CXCR1 expression was extremely low in breast cancer cells, whereas most of the cells investigated showed a higher expression of CXCR2 [52]. The same study also suggested that IL-8 expression is negatively correlated to ER-status and is expressed preferentially in invasive cancer cells [52]. Moreover, our data showed that the higher percentage of ER-negative tumors were present in patients carrying the *IL-8 (-251) A *allele which is associated with a higher IL-8 production. Altogether, these results suggested that the genetic variation in the promoter of the *IL-8 *gene which influences the production of this chemokine could be the genetic basis of the potential tumor progression and invasiveness of breast cancer.

The ability of IL-8 to elicit angiogenic activity depends on the endothelial cell expression of its receptors. Recent studies indicated that CXCR1 and CXCR2 are highly expressed on human microvascular endothelial cells (HMEC) [53]. Antibodies directed at CXCR1 and CXCR2 are capable of inhibiting IL-8 induced migration of HMEC, which indicates that these two receptors are critical for the IL-8 angiogenic response. Since CXCR2 binds to all ELR^+^CXC chemokines that induce angiogenesis, including IL-8, it may be safe to say that CXCR2 is a mediator of the proangiogenic effects of IL-8.

Several reports have confirmed the importance of CXCR2 in mediating the effects of angiogenesis in human microvascular endothelial cells [18,36,53]. Endothelial cells were found to express CXCR2 *in vitro *and *in vivo*, but not CXCR1. Blocking the function of CXCR2 by either neutralizing antibodies or inhibiting downstream signalling using specific inhibitors of ERK1/2 and PI3 kinase impaired IL-8-induced stress fiber assembly, chemotaxis, and endothelial tube formation in endothelial cells [39,54]. Overall, these data strongly support a role for CXCR2 in angiogenesis induced by ELR^+^CXC chemokines and especially by IL-8.

## Conclusion

In summary, our data suggests that *IL-8 *(-251) T/A and *CXCR2 *(+1208) C/T polymorphisms are likely to play a major role in susceptibility to and prognosis in breast carcinoma. Although additional studies on a larger scale will be required to confirm and extend our findings, the present data suggest for the first time that *CXCR2 *(+1208) C/T polymorphism represents a risk factor for poorer prognosis and susceptibility to breast carcinoma. Furthermore, this study provides support for the multigenetic effects of the variant alleles from *IL-8*, and *CXCR2*, resulting in a significantly increased risk for breast cancer in the Tunisian population. Our findings also reinforce the role attributed to inflammation, angiogenesis, and their mediators as major contributing factors in the process of breast tumor development, progression and aggressiveness. Using the *IL-8 *(-251) T/A and *CXCR2 *(+1208) C/T polymorphisms alone or in combination with other genetic polymorphisms in angiogenic and inflammatory genes to predict breast carcinoma outcome and prognosis may therefore have an important clinical significance.

## Competing interests

The authors declare that they have no competing interests.

## Authors' contributions

SK conceived the manuscript, conducted data analysis, and drafted the manuscript. MW contributed to the design and management of data. HK, MF and NB provided samples and clinical information. LC designed and participated in the data analysis and interpretation of the study. AN-H contributed to reviewing the manuscript. All authors read and approved the final manuscript.

## Pre-publication history

The pre-publication history for this paper can be accessed here:

http://www.biomedcentral.com/1471-2407/10/283/prepub
